# Do NIHR health technology assessment randomised clinical trials perform as well as expected?

**DOI:** 10.1186/1745-6215-14-S1-P92

**Published:** 2013-11-29

**Authors:** Amanda Young, James Raftery, Louise Stanton, Andrew Cook

**Affiliations:** 1 National Institute for Health Research, Evaluation, Trials and Studies Coordinating Centre, Southampton, UK; 2 Clinical Trials Unit, University of Southampton, Southampton, UK

## Background

It is widely reported that clinical trials often experience delays and make changes to the pre-specified protocol. Campbell *et al.* (2007) found less the one third of UK publicly funded studies recruited according to plan.

## Objectives

To assess how well published HTA clinical trials perform, including recruitment patterns, frequency and type of protocol changes, extension request approvals and amendments to the sample size calculation.

## Methods

All randomised clinical trials published in the HTA Journal Series between 1999 and 2011. The unit of analysis was the clinical trial funded by the HTA Programme. Pre-defined protocols were used to determine the ‘expected' performance, whilst published reports were used to determine whether those plans were met. Data were extracted into the main study metadata Access database.

## Results

125 clinical trials published in the HTA Journal Series met the inclusion. Five trials were reported to have been abandoned and were excluded from analyses. 72% of clinical trials achieved 50% or better of their original recruitment target. 90% of clinical trials recruited their targeted number of centres with 50% recruiting more than the initial number they expected.

One third of trials amended the sample size calculation after the trial commenced with more than 80% decreasing the number of participants needed for the trial.

## Conclusions

Reviewing the quality of performance of clinical trials will provide important feedback to research management centres about how realistic clinical trialists need to be about trial set up times and its impact on the time required to recruit participating centres.

